# Efficacy and safety of Chinese medicines for asthma: A systematic review protocol

**DOI:** 10.1097/MD.0000000000016958

**Published:** 2019-08-23

**Authors:** Qi Shi, Dongxu Si, Haipeng Bao, Yue Yan, Yanhua Kong, Chunlei Li, Wenfeng He, Dashzeveg Damchaaperenlei, Mingxia Yu, Youlin Li

**Affiliations:** a The 2nd Department of Pulmonary Disease in TCM, The Key Unit of SATCM Pneumonopathy Chronic Cough and Dyspnea, Beijing Key Laboratory of Prevention and Treatment of Allergic Diseases with TCM (No. BZ0321), Center of Respiratory Medicine, China-Japan Friendship Hospital, National Clinical Research Center for Respiratory Diseases; b Beijing University of Chinese Medicine, Beijing; c Inner Mongolia Autonomous Region Hospital of Traditional Chinese Medicine, Hohhot, China.

**Keywords:** chronic duration of asthma, protocol, systematic review, traditional Chinese medicines

## Abstract

**Background:**

Asthma is a complex disease associated with many factors such as immunologic, environmental, genetic, and other factors. Common medicines used to treat asthma include β-agonist and glucocorticoid. However, in the long-term treatment, the effect of the above-mentioned drugs is not satisfactory, so many patients choose oral Chinese medicines instead of western medicines. The introduction of Chinese medicines therapies, a rapid proliferation of the literature on management of asthma in general, call for novel ways of evidence synthesis in this area. This systematic review is to systematically summarize and evaluate a large number of evidences for Chinese herbal interventions for asthma. Evaluate the efficacy and safety of Chinese medicines in the treatment of asthma and inform a decision aid for the clinical encounter between patients and clinicians. In addition, it helps to establish a future research agenda.

**Methods:**

Five English databases (PubMed, Web of science, EBASE, Springer Cochrane Library, and WHO International Clinical Trials Registry Platform) and 4 Chinese databases (Wanfang Database, Chinese Scientific Journal Database, China National Knowledge Infrastructure Database, and Chinese Biomedical Literature Database) will be searched normatively according to the rule of each database from the inception to the present. The literature screening, data extraction, and quality assessment will be conducted by 2 researchers independently. Data will be synthesized by either the fixed-effects or random-effects model according to a heterogeneity test. Asthma control test symptom score will be assessed as the primary outcome. The curative effect of single symptom and sign; Withdrawal and reduction of western medicines in a course of treatment, including: time, type, and quantity; Maintenance of western medicines after the course of treatment, including: type, quantity; Asthma Quality of Life Questionnaire; laboratory efficacy indexes as the secondary outcome. General physical examination; routine examination of blood, urine, and stool; electrocardiogram; liver and kidney function examination; possible adverse reactions and related detection indicators as the security indexes. Meta-analysis will be performed using RevMan5.3.5 software provided by the Cochrane Collaboration.

**Results:**

This study will provide high-quality synthesis based on current evidence of Chinese medicines treatment for asthma in several aspects, including asthma control score, side effects and laboratory examination such as lung-function test, serum total immunoglobulin, and so on.

**Conclusion:**

The results of this study will provide updated evidence for whether Chinese medicines is an effective and safe intervention for asthma.

**PROSPERO registration number:**

PROSPERO CRD42019136074.

## 1 Introduction

Asthma, affecting 1% to 18% of the population in different countries, is a complex disease associated with many factors such as immunologic, environmental, genetic, and other factors. It usually characterized by chronic airway inflammation and defined by the history of respiratory symptoms such as wheeze, shortness of breath, chest tightness, and cough that vary over time and in intensity, together with variable expiratory airflow limitation.^[1]^ Chronic inflammation of the airways recognized to be controlled by the T-helper 2 (Th2) lymphocytes, which secrete cytokines to increase the production of immunoglobulin (IgE) by B cells.^[2]^ Eosinophils (EOS) is one of the most important cells involved in the pathogenesis of chronic airway inflammation in asthma.^[3]^

Asthma can be divided into acute and nonacute attack period. Acute attack period, clinicians advocate the use of Western medicines (such as bronchodilators,^[4]^ monoclonal antibodies,^[5,6]^ corticosteroids,^[7,8]^ the combination of inhaled corticosteroid and long-acting β2-agonist,^[9]^ and others^[10]^), even bronchial thermoplasty^[11]^ symptomatic supportive treatment to relieve symptoms. Most patients may benefit from these treatments. Nonacute attack period, also known as chronic duration, refers to the patient although there is no acute attack of asthma, but in a considerable period of time still have different frequency and different degrees of wheezing, coughing, chest tightness, and other symptoms. Thus, due to the persistence of symptoms in chronic duration asthma, and the above-mentioned drugs treatment is not satisfactory, Patients’ conditions were not effectively controlled and their quality of life was not improved. In addition, some patients were unable to tolerate the above drugs^[12–14]^ or show side effects, such as gastrointestinal, cardiovascular, metabolic, and bone-related complications that have been reported from long-term use of the above-mentioned medications.^[15–17]^ Therefore, many patients choose Chinese medicines, with relatively few side effects, to treat asthma. Traditional Chinese medicine (including Chinese medicines,^[18,19]^ acupuncture,^[20]^ massage, acupoint sticking therapy,^[21]^ acupoint catgut embedding therapy,^[22]^ diet therapy,^[23]^ and other physical interventions) is widely applied for asthma.

Among the above treatment measures, Patients often choose oral Chinese medicines to treat asthma. Many clinical and experimental studies have confirmed the effectiveness of Chinese medicines in the treatment of asthma, such as Wentong decoction could accelerate EOS apoptosis, reduce asthma inflammation, and alleviate the disease through regulating and controlling the factors related to the anti-apoptosis and pro-apoptosis^[3]^; Yupingfeng San could inhibit NOD-like receptor family pyrin domain-containing 3 inflammasome components to attenuate the inflammatory response in asthma.^[24]^ Many Chinese herbs could inhibit the activation and migration of inflammatory cells, regulate the balance of Th1/Th2 responses, and suppress allergic hyperreactivity through inducing regulatory T cells or attenuating the function of dendritic cells.^[25]^ Pingchuan formula and Bu-Shen-Yi-Qi formulae could improves asthma via restoration of the Th17/Treg balance in a mouse model.^[26,27]^

Although a rapid proliferation of the literature on the introduction of Chinese medicines therapies of asthma in general, few systematic analyses and syntheses are identified. Thus, call for novel ways of evidence synthesis in this area. This systematic review is to systematically summarize and evaluate a large number of evidences for Chinese herbal interventions for asthma. Evaluate the efficacy and safety of Chinese medicines in the treatment of asthma and inform a decision aid for the clinical encounter between patients and clinicians. In addition, it helps to establish a future research agenda.

## 2 Methods

### 2.1 Study registration

The protocol of this review has been registered with the International Prospective Register of Systematic Reviews (PROSPERO; http://www.crd.york.ac.uk/PROSP-ERO/display_record.php?ID=CRD42019136074) and has been reported in accordance with the Preferred Reporting Items for Systematic Reviews and Meta-analyses guidelines.^[28]^

### 2.2 Inclusion criteria for study selection

#### 2.2.1 Types of studies

In order to ensure the quality of this systematic review, we will include all the relevant randomized controlled trials (RCTs) published in English or Chinese for the treatment of asthma. The current clinical trial results will be objectively integrated, which is conducive to the evaluation of the efficacy and safety of Chinese medicines in the treatment of asthma.

Non-RCTs, cohort studies, reviews, case reports, experimental studies, expert experience, the data of the included study is missing or incomplete, and duplicate publications will be excluded.

#### 2.2.2 Types of participants

Asthma participants of all ages will be included in the study regardless of nationality, gender, race, occupation, or education level.

Patients with drug-induced asthma, aspirin intolerance triad, asthma with fixed airflow limitation, asthma with obesity, late-onset asthma will be excluded.

#### 2.2.3 Types of interventions

This study focuses on the clinical trial (RCTs) of asthma with the therapy of Chinese medicines, and the results will provide advice and consultation for clinicians. Therefore, patients in the experimental group were only treated with Chinese medicines, and the types and dosage forms of Chinese medicines prescriptions were not limited. In addition, western medicines and other treatment methods were not combined (except for emergency medical use). Studies that with combination therapy fail to objectively evaluate the efficacy and safety of Chinese medicines will be excluded.

Studies of control groups were treated with western medicines or placebo and other interventions (eg, acupuncture, moxibustion, and other physical interventions).

#### 2.2.4 Types of outcome measures

##### 2.2.4.1 Primary outcomes

Asthma control test^[29]^ symptom score was assessed as the primary outcome. Symptom score: Step 1: accurately record the score of each question; Step 2: add up the scores of each question to get the total score; Step 3: find the meaning of the total score (3 levels: 25 points: total control; 20–24 points: partial control; <20 points: not under control).

##### 2.2.4.2 Secondary outcomes

The secondary outcomes of this review mainly include the following aspects:

(1) The curative effect of single symptom and sign.
(2) Withdrawal and reduction of western medicines in a course of treatment, including: time, type and quantity; maintenance of western medicines after the course of treatment, including: type, quantity.
(3) Asthma Quality of Life Questionnaire.^[9,30]^
(4) Laboratory efficacy indexes.
  (1) Serum CD4^+^, CD8^+^ levels, and CD4^+^/CD8^+^ ratio.
  (2) The Th1, Th2.^[25]^
  (3) Serum IgE.^[31]^
  (4) Blood eosinophil count.^[9]^
  (5) Skin prick test.

##### 2.2.4.3 Security index

(1) General physical examination (temperature, pulse, respiration, blood pressure).
(2) Routine examination of blood, urine, and stool.
(3) Electrocardiogram.
(4) Liver and kidney function examination.
(5) Possible adverse reactions and related detection indicators.

### 2.3 Search methods for identification of studies

#### 2.3.1 Electronic searches

Five English databases (PubMed, Web of science, EBASE, Springer Cochrane Library, and WHO International Clinical Trials Registry Platform) and 4 Chinese databases (Wanfang Database, Chinese Scientific Journal Database, China National Knowledge Infrastructure Database, and Chinese Biomedical Literature Database) will be searched normatively according to the rule of each database from the inception to the present.

#### 2.3.2 Searching strategy

The search strategy for PubMed is listed in Table 1, which including all search terms, and other searches will be conducted based on these results. This search strategy will be modified as required for other electronic databases.

**Table 1:**
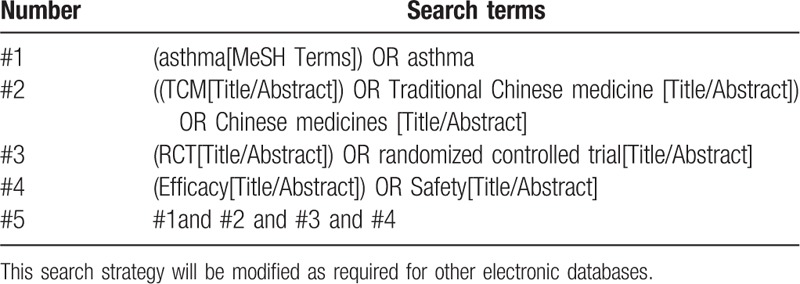
The search strategy for PubMed.

### 2.4 Data collection and analysis

#### 2.4.1 Selection of studies

**Figure 1:**
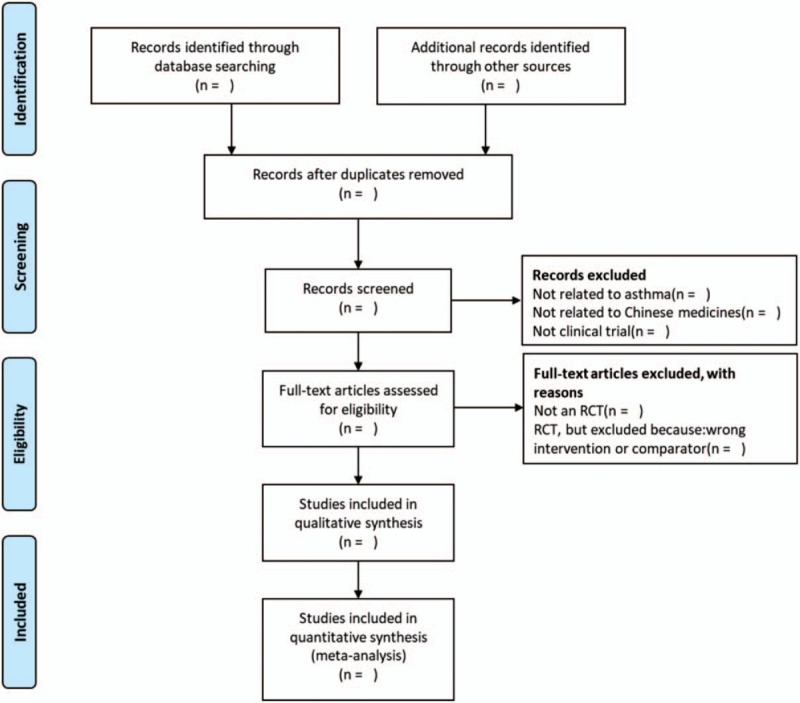
The PRISMA flow chart of the selection process. PRISMA = preferred reporting items for systematic reviews and meta-analysis protocol.

The basic process of the included literatures will be determined by referring to the Cochrane Collaboration System Evaluator's Manual (5.1.0). The study name, author, year, database, and whether the study met eligibility criteria and should be included in the review will be recorded using an excel spreadsheet. Reasons for inclusion and exclusion (PICOS) are documented in a spreadsheet during abstract screening and full-text evaluation. The records in this spreadsheet will be used to generate the Preferred Reporting Items for Systematic Reviews and Meta-Analyses flowchart (Fig. 1). All the procedures will be carried out by 2 independent reviewers and completed cross-check. If there is any disagreement, the 3rd author will be invited to assist in the discussion and make a decision.

#### 2.4.2 Unit of analysis issues

We will only extract the 1st experimental period data of crossover trials to avoid carryover effects. With multiple intervention groups, we will combine all relevant experimental groups and control intervention groups into a single respectively group to prevent a unit of analysis issue.

#### 2.4.3 Data extraction and management

Two independent reviewers will extract the data of interest from the eligible study and enter the following information in the data extraction sheet: The basic characteristics of each study (1st author, title, source/journal, time of publication, and country); participants characteristics (average age, gender, sample size, inclusion and exclusion criteria, baseline situation); Interventions (type of Chinese medicines, randomization, allocation concealment, and blinding methods); comparators (western medicines); outcomes (measures, main outcomes, security indexes, and follow up); if funded, it will also be recorded. When the consensus on data extraction is not available through discussion, the third reviewer will make a decision.

#### 2.4.4 Data synthesis

RevMan5.3.5 will be used for all statistical analyses. Based on the heterogeneity levels of the included studies, a fixed-effects model will be used if there is no significant heterogeneity of the data (*I*^2^ ≤ 50%); a random-effects model will be used if significant heterogeneity existed (*I*^2^ > 50%). The dichotomous data will be analyzed by risk ratio with 95% confidence intervals (CIs), while the continuous data will be analyzed by mean difference (MD) or standard MD with 95% CIs. The meaningful heterogeneity will be explained by any additional assessment included sensitivity analysis or subgroup analysis depended on the data.

#### 2.4.5 Assessment of study quality

A rigorous quality assessment of the literatures included in the study will be conducted using the bias risk assessment tool (RevMan5.3.5) of the Cochrane Collaborative Network System, and high, low, unclear evaluations will be performed for each item. Meanwhile, the STRICTA checklist will be completed. When 2 quality assessors are unable to reach a consensus on the risk assessment by negotiation, the third the third reviewer will make a decision.

#### 2.4.6 Assessment of reporting bias

When 10 or more studies are included in a meta-analysis, we will assess the reporting bias. The trim and fill method will be used to identify and correct for funnel plot asymmetry arising from publication bias, if appropriate.^[32]^

#### 2.4.7 Assessment of heterogeneity

Data analysis will be performed using RevMan5.3.5 software provided by the Cochrane Collaboration. Using the software to obtain forest plots and test the heterogeneity between the included studies. Chi-square test and *I*^2^ value will be applied to calculate and present the heterogeneity degree. When *P* > .1, *I*^2^ < 50%, it is considered that there is no heterogeneity between the trials, and the fixed effect model will be used for statistics, otherwise, the random effect model will be adopted to analyze.

#### 2.4.8 Subgroup analysis

When heterogeneity is detected, we will judge the source of heterogeneity through subgroup analysis (eg, different types of Chinese medicines therapies, research quality, publication age, participation population, length of treatment). In addition, we can also observe the relationship between effect values and grouping variables.

#### 2.4.9 Sensitivity analysis

In the case of sufficient trials data, sensitivity analysis is mainly carried out for research characteristics or types such as methodological quality, and the effect on total effects is examined by excluding certain low-quality studies or small-sample sizes of studies. When heterogeneity occurs, we may perform sensitivity analysis by excluding the data one by one.

## 3 Discussion

Asthma can be divided into acute and nonacute attack period. In the acute exacerbation period, β-agonist and glucocorticoid are used to symptomatic treatment. Non-acute attack period, due to the persistence of symptoms, and the above-mentioned drugs treatment is not satisfactory. Patients’ conditions were not effectively controlled and their quality of life was not improved. Furthermore, many patients were unable to tolerate the above drugs or show side effects. Therefore, many patients choose oral Chinese medicines instead of western medicines. Although Chinese medicines have a certain unique therapeutic effect on asthma. However, due to the complex composition of Chinese medicines, potential safety hazards may exist. The purpose of this systematic review is to systematically summarize and evaluate a large number of evidences for Chinese herbal interventions for asthma. Evaluate the efficacy and safety of Chinese medicines in the treatment of asthma and inform a decision aid for the clinical encounter between patients and clinicians. In addition, it helps to establish a future research agenda.

## Author contributions

**Conceptualization:** Qi Shi, Dongxu Si, Youlin Li.

**Data curation:** Haipeng Bao, Wenfeng He, Dashzeveg Damchaaperenlei, Mingxia Yu.

**Formal analysis:** Dongxu Si, Haipeng Bao.

**Project administration:** Qi Shi, Dongxu Si, Haipeng Bao.

**Supervision:** Qi Shi, Yue Yan, Yanhua Kong, Chunlei Li.

**Writing – original draft:** Qi Shi, Dongxu Si.

**Writing – review and editing:** Qi Shi, Youlin Li.

## References

[1] Global Initiative for Asthma. Global Strategy for Asthma Management and Prevention, 2018 Available at: www.ginasthma.org

[2] Ayakannu R Abdullah NA Radhakrishnan AK Relationship between various cytokines implicated in asthma. Hum Immunol 2019;pii:S0198-8859(19)30010-2. 10.1016/j.humimm.2019.04.018 31054782

[3] Yan Y Bao HP Li CL Wentong decoction cures allergic bronchial asthma by regulating the apoptosis imbalance of EOS. Chin Med 2018;13:21. 29713367 10.1186/s13020-018-0180-2 PMC5907368

[4] Cazzola M Rogliani P Calzetta L Bronchodilators in subjects with asthma-related comorbidities. Respir Med 2019;151:43–8. 31047116 10.1016/j.rmed.2019.04.001

[5] Koski RR Grzegorczyk KM Comparison of monoclonal antibodies for treatment of uncontrolled eosinophilic asthma. J Pharm Pract 2019;2:897190019840597. 10.1177/0897190019840597 31046541

[6] Canonica GW Rottoli P Bucca C Improvement of patient-reported outcomes in severe allergic asthma by omalizumab treatment: the real life observational PROXIMA study. World Allergy Organ J 2018;11:33. 30410639 10.1186/s40413-018-0214-3 PMC6214174

[7] O’Byrne P Fabbri LM Pavord ID Asthma progression and mortality: the role of inhaled corticosteroids. Eur Respir J 2019;54:1900491. 31048346 10.1183/13993003.00491-2019 PMC6637285

[8] Prazma CM Bel EH Price RG Oral corticosteroid dose changes and impact on peripheral blood eosinophil counts in patients with severe eosinophilic asthma: a post hoc analysis. Respir Res 2019;20:83. 31053134 10.1186/s12931-019-1056-4 PMC6499981

[9] Zairina E Nugraheni G Achmad GN Efficacy of an education session by pharmacists for patients with asthma: protocol and design of a randomized controlled trial. JMIR Res Protoc 2018;7:e10210. 30563816 10.2196/10210 PMC6315257

[10] Glady G Clinical efficacy of implementing Bio Immune(G)ene MEDicine in the treatment of chronic asthma with the objective of reducing or removing effectively corticosteroid therapy: a novel approach and promising results. Exp Ther Med 2018;15:5133–40. 29805540 10.3892/etm.2018.6019 PMC5952088

[11] Seeley EJ Alshelli I Canfield J The impact of bronchial thermoplasty on asthma-related quality of life and controller medication use. Respiration 2019;2:1–6. 10.1159/000499404 31048594

[12] Yoshihara S Tsubaki T Ikeda M The efficacy and safety of fluticasone/salmeterol compared to fluticasone in children younger than four years of age. Pediatr Allergy Immunol 2019;30:195–203. 30556939 10.1111/pai.13010 PMC6850202

[13] Dusser D Ducharme FM Safety of tiotropium in patients with asthma. Ther Adv Respir Dis 2019;13:1753466618824010. 30795731 10.1177/1753466618824010 PMC6391545

[14] Katsaounou P Buhl R Brusselle G Omalizumab as alternative to chronic use of oral corticosteroids in severe asthma. Respir Med 2019;150:51–62. 30961951 10.1016/j.rmed.2019.02.003

[15] Bloechliger M Reinau D Spoendlin J Adverse events profile of oral corticosteroids among asthma patients in the UK: cohort study with a nested case-control analysis. Respir Res 2018;19:75. 29699563 10.1186/s12931-018-0742-y PMC5921395

[16] Tang Y Zhang C Zhang Z The efficacy and safety of different long-acting beta2-agonists combined with inhaled glucocorticoid regimens in patients with asthma: a network meta-analysis. J Asthma 2018;25:1–3. 10.1080/02770903.2018.1531991 30359144

[17] Price DB Trudo F Voorham J Adverse outcomes from initiation of systemic corticosteroids for asthma: long-term observational study. J Asthma Allergy 2018;11:193–204. 30214247 10.2147/JAA.S176026 PMC6121746

[18] Huang TP Liu PH Lien AS Characteristics of traditional Chinese medicine use in children with asthma: a nationwide population-based study. Allergy 2013;68:1610–3. 24117783 10.1111/all.12273

[19] Zhao YH Liu ZI Li LH Systematic review of randomized controlled trials of traditional Chinese medicine treatment of non-acute bronchial asthma complicated by gastroesophageal reflux. J Tradit Chin Med 2012;32:12–8. 22594096 10.1016/s0254-6272(12)60025-9

[20] Wen CY Liu YF Zhou L A systematic and narrative review of acupuncture point application therapies in the treatment of allergic rhinitis and asthma during dog days. Evid Based Complement Alternat Med 2015;2015:846851. 26543488 10.1155/2015/846851 PMC4620249

[21] Yao H Tong J Zhang PD Acupoint sticking therapy for treatment of bronchial asthma: a multicenter controlled randomized clinical trial. Zhongguo Zhen Jiu 2009;29:609–12. 19947260

[22] Chen PB Cui J Yang XF Controlled study on different acupoint-prescription for the acupoint catgut embedding therapy in treatment of bronchial asthma. Zhongguo Zhen Jiu 2012;32:630–3. 22997795

[23] Deng Z TCM diet therapy for bronchial asthma. J Tradit Chin Med 2009;29:209–10. 19894387 10.1016/s0254-6272(09)60067-4

[24] Liu X Shen J Fan D Yupingfeng San inhibits NLRP3 inflammasome to attenuate the inflammatory response in asthma mice. Front Pharmacol 2017;8:944. 29311942 10.3389/fphar.2017.00944 PMC5743824

[25] Li J Zhang F Li J The immunoregulatory effects of traditional Chinese medicine on treatment of asthma or asthmatic inflammation. Am J Chin Med 2015;43:1059–81. 26364661 10.1142/S0192415X15500615

[26] Liu F Yu J Bai L Pingchuan formula improves asthma via restoration of the Th17/Treg balance in a mouse model. BMC Complement Altern Med 2015;15:234. 26178910 10.1186/s12906-015-0755-8 PMC4502614

[27] Wei Y Luo QL Sun J Bu-Shen-Yi-Qi formulae suppress chronic airway inflammation and regulate Th17/Treg imbalance in the murine ovalbumin asthma model. J Ethnopharmacol 2015;164:368–77. 25625352 10.1016/j.jep.2015.01.016

[28] Moher D Shamseer L Clarke M Preferred reporting items for systematic review and meta-analysis protocols (PRISMA-P) 2015 statement. Syst Rev 2015;4:1. 25554246 10.1186/2046-4053-4-1 PMC4320440

[29] Harris K Mosler G Grigg J Theory-based self-management intervention to improve adolescents’ asthma control: a cluster randomised controlled trial protocol. BMJ Open 2019;9:e25867. 10.1136/bmjopen-2018-025867 PMC6500249 31015270

[30] Shi X Buysse DJ Ritterband LM Solving insomnia electronically: sleep treatment for asthma (SIESTA): a study protocol for a randomized controlled trial. Contemp Clin Trials 2019;79:73–9. 30825525 10.1016/j.cct.2019.02.011 PMC6563833

[31] Lux H Lenz K Budnik LT Performance of specific immunoglobulin E tests for diagnosing occupational asthma: a systematic review and meta-analysis. Occup Environ Med 2019;76:269–78. 30804164 10.1136/oemed-2018-105434

[32] Vandenbroucke JP Bias in meta-analysis detected by a simple, graphical test. Experts’ views are still needed. BMJ 1998;316:469–70. PMC2665608 9492686

